# Intercomparison of MR‐informed PET image reconstruction methods

**DOI:** 10.1002/mp.13812

**Published:** 2019-10-04

**Authors:** James Bland, Abolfazl Mehranian, Martin A. Belzunce, Sam Ellis, Casper da Costa‐Luis, Colm J. McGinnity, Alexander Hammers, Andrew J. Reader

**Affiliations:** ^1^ School of Biomedical Engineering and Imaging Sciences King's College London St Thomas' Hospital London SE1 7EH UK; ^2^ King's College London & Guy's and St Thomas' PET Centre St Thomas' Hospital London SE1 7EH UK

**Keywords:** kernel, MAPEM, MR-informed, PET, reconstruction

## Abstract

**Purpose:**

Numerous image reconstruction methodologies for positron emission tomography (PET) have been developed that incorporate magnetic resonance (MR) imaging structural information, producing reconstructed images with improved suppression of noise and reduced partial volume effects. However, the influence of MR structural information also increases the possibility of suppression or bias of structures present only in the PET data (PET‐unique regions). To address this, further developments for MR‐informed methods have been proposed, for example, through inclusion of the current reconstructed PET image, alongside the MR image, in the iterative reconstruction process. In this present work, a number of kernel and maximum *a posteriori* (MAP) methodologies are compared, with the aim of identifying methods that enable a favorable trade‐off between the suppression of noise and the retention of unique features present in the PET data.

**Methods:**

The reconstruction methods investigated were: the MR‐informed conventional and spatially compact kernel methods, referred to as KEM and KEM largest value sparsification (LVS) respectively; the MR‐informed Bowsher and Gaussian MR‐guided MAP methods; and the PET‐MR‐informed hybrid kernel and anato‐functional MAP methods. The trade‐off between improving the reconstruction of the whole brain region and the PET‐unique regions was investigated for all methods in comparison with postsmoothed maximum likelihood expectation maximization (MLEM), evaluated in terms of structural similarity index (SSIM), normalized root mean square error (NRMSE), bias, and standard deviation. Both simulated BrainWeb (10 noise realizations) and real [^18^F] fluorodeoxyglucose (FDG) three‐dimensional datasets were used. The real [^18^F]FDG dataset was augmented with simulated tumors to allow comparison of the reconstruction methodologies for the case of known regions of PET‐MR discrepancy and evaluated at full counts (100%) and at a reduced (10%) count level.

**Results:**

For the high‐count simulated and real data studies, the anato‐functional MAP method performed better than the other methods under investigation (MR‐informed, PET‐MR‐informed and postsmoothed MLEM), in terms of achieving the best trade‐off for the reconstruction of the whole brain and PET‐unique regions, assessed in terms of the SSIM, NRMSE, and bias vs standard deviation. The inclusion of PET information in the anato‐functional MAP method enables the reconstruction of PET‐unique regions to attain similarly low levels of bias as unsmoothed MLEM, while moderately improving the whole brain image quality for low levels of regularization. However, for low count simulated datasets the anato‐functional MAP method performs poorly, due to the inclusion of noisy PET information in the regularization term. For the low counts simulated dataset, KEM LVS and to a lesser extent, HKEM performed better than the other methods under investigation in terms of achieving the best trade‐off for the reconstruction of the whole brain and PET‐unique regions, assessed in terms of the SSIM, NRMSE, and bias vs standard deviation.

**Conclusion:**

For the reconstruction of noisy data, multiple MR‐informed methods produce favorable whole brain vs PET‐unique region trade‐off in terms of the image quality metrics of SSIM and NRMSE, comfortably outperforming the whole image denoising of postsmoothed MLEM.

## 1 Introduction

Positron emission tomography (PET) is a versatile and clinically impactful medical imaging modality, in the diagnosis or management of neurological disorders, cancers, and cardiovascular diseases. Despite PET's clinical utility, the associated image quality is generally inferior to other anatomically driven imaging modalities, such as magnetic resonance imaging (MRI). There are two dominant factors that degrade the reconstructed PET image quality: (a) high Poisson noise in the PET data, a consequence of the limited injected dose and limited sensitivity of current clinical PET scanners; (b) poor spatial resolution (~4 mm), due to the finite detector size, photon acollinearity and positron range (among other factors). The limited PET system resolution leads to partial volume effects (PVEs), such as the spillover of small high‐intensity regions to neighboring voxels, introducing bias in regional quantification. To address these issues, anatomical images (such as MR) have been exploited, through utilizing shared PET‐MR structural information within the reconstruction process. Anatomical MR images, such as T1 or T2 weighted, provide structural information that the PET radiotracer distribution is likely to correlate with, at least in part. This functional anatomical correspondence is particularly evident for neurological [^18^F]fluorodeoxyglucose (FDG) PET scans, where the radiotracer distribution is well delineated between the white and gray matter boundaries. Numerous such MR‐informed reconstruction methodologies have now been proposed in the literature.^1^, ^2^, ^3^, ^4^, ^5^, ^6^, ^7^, ^8^

One recent example of MR‐informed PET reconstruction in the current literature is the MR‐informed kernel method (KEM).^9^, ^10^ The kernel method extracts structural information from the corresponding MR image, forming a set of spatial basis functions. These structural basis functions reparameterize the reconstruction process, restricting the reconstructed image to be comprised from a linear combination of these spatial basis functions. MR‐informed KEM has subsequently been extended to produce more spatially compact basis functions, referred to as KEM largest value sparsification (LVS),^11^ to aid the recovery of PET‐unique features. PET‐unique features are considered to be structures that are present in the PET image, but which are not present or have a different structure in the MR image. In contrast to conventional KEM, KEM LVS uses a composite feature vector comprised of both MR intensity and spatial information, the relative weighting between these two features is used to select the contributing voxels for each basis function. Therefore, in relatively uniform MR regions where the MR intensity values are similar, spatially close voxels will be selected over more disparate voxels, thereby helping recovery of PET‐unique features. More generally, KEM has been applied to a range of reconstruction problems,^12^, ^13^, ^14^, ^15^, ^16^, ^17^, ^18^, ^19^, ^20^, ^21^ and is an example of a broader cohort of algorithms that reparameterize the emission image into an alternative set of basis functions.^22^, ^23^, ^24^

In contrast to reparameterizing the reconstruction process, MR information can alternatively be included into the reconstruction process through the addition of a regularizing term in either a Bayesian maximum *a posteriori* (MAP) or penalized maximum likelihood (PL) framework.^25^, ^26^ In this work, the regularized methods investigated are restricted to an image‐weighted quadratic potential function in the prior. Multiple alternatives to the quadratic prior have been proposed in the literature (e.g., relative difference, total variation, Lange, Kaipio),^27^, ^28^, ^29^ which predominantly seek to reduce smoothing across genuine boundaries in comparison with the quadratic prior. However, due to the inclusion of anatomical boundary information through the image‐weighting factors, shared PET‐MR boundaries are expected to be well recovered irrespective of the potential function used in the prior. For such reasons, the consequence of varying penalty functions (for instance using the relative difference instead of the quadratic penalty function) while including image‐weighting factors in the prior has been shown to be minimal.^30^

The selection of MR(only)‐informed weighting factors under comparison in this work is the Gaussian similarity kernel^31^, ^32^ and the asymmetric Bowsher prior,^5^ due to their enduring popularity, and ability to match the performance of more involved MR‐informed methods.^30^, ^33^, ^34^ In addition, these methods use spatial similarity matrices to extract MR structures, in a comparable way to the kernel methods. Thus, all methods investigated in this study can be considered to be part of the same group of algorithms which include MR information through similarity matrices. These MR‐derived similarity matrices are incorporated into the reconstruction process via the kernel method (KEM & KEM LVS) or MAP (Gaussian MR‐Guided and Bowsher).

The kernel and MAP MR‐informed reconstruction methodologies have all demonstrated reduced noise^35^ and reduced PVE properties, in comparison with the routinely used maximum likelihood expectation maximization (MLEM)^36^ or ordered subsets expectation maximization (OSEM) algorithms.^37^ Due to the reduction of PVE through incorporating MR information, major improvements in regional quantification can be realized.^38^, ^39^ This is of particular importance for the assessment and diagnosis of neurological diseases including Alzheimer's, epilepsy, and Parkinson's disease, where quantification of MR visible anatomical regions is essential.^6^, ^40^, ^41^, ^42^, ^43^, ^44^ Despite these beneficial properties achieved through the inclusion of MR information, adverse consequences also arise, such as increased susceptibility to suppressing PET‐unique high‐intensity regions.^10^, ^30^ This is a major pitfall for the visual diagnostic interpretation of PET images, in particular for oncological cases, where PET imaging has been shown to improve the diagnosis and subsequent treatment of cancers.^45^ Specifically, for brain and neck cancers [^18^F]FDG PET has played an increasing role in the diagnosis and planning.^46^, ^47^ One potential avenue currently under investigation for reducing the suppression of these high‐intensity PET‐unique regions is to extend the MR guidance to include the reconstructed PET image at each iteration. This concept was implemented firstly via MAP (regularization)^3^, ^48^, ^49^ and has recently been extended to a kernel (reparameterization)^50^, ^51^ implementation. Such methods shall be referred to as PET‐MR‐informed, from which this work shall compare the anato‐functional (a MAP method),^52^ and the hybrid kernel method (HKEM).^50^, ^51^ Alternative PET‐MR‐informed methods are present in the literature, which incorporate both PET and MR information in the regularization term, including joint Shannon entropy^4^, ^53^ and parallel level sets priors.^54^, ^55^ While such methods remain active within the PET reconstruction field, they do not lie within the same group of algorithms under comparison that determine PET‐MR similarity via a local neighborhood similarity matrix.

In this work, a comparison between MR‐informed and PET‐MR‐informed kernel and MAP methods is undertaken, in order to clarify whether the inclusion of MR information into the PET reconstruction process in the above forms can universally improve the reconstructed images, for shared and discrepant PET‐MR regions alike. In particular, we focus on the trade‐off between whole brain image quality, evaluated in terms of structural similarity (SSIM) and normalized root mean square error (NRMSE), and the faithful recovery of structures unique to the PET data, evaluated in terms of bias and mean values, and whether the inclusion of PET data into the guidance process reduces the suppression of the PET‐unique regions.

## 2 Materials and Methods

### 2.1 Theory

The reconstructed PET image represents the spatial distribution of an injected radiotracer. The injected radiotracer undergoes radioactive decay within the patient, expelling positrons that rapidly annihilate, with each annihilation event producing a pair of photons with opposite trajectories that are detected by the PET scanner along a particular line of response. The measured counts (***m***) for each and every line of response correspond to a set of Poisson random variables, the values of which can be related to the expected counts through the Poisson likelihood. Through maximizing the Poisson likelihood with respect to the emission image (***θ***), the most likely expected counts distribution is calculated. The EM algorithm is a popular iterative method to maximize the Poisson likelihood objective function^56^:

(1) $$\begin{array}{c} L\left(q\left(\theta \right);m\right)=\sum_{i}m_{i}\log\left(q_{i}\right)-q_{i} \end{array}$$

(2) $$\begin{array}{c} q\left(\theta \right)=A\theta +b \end{array}$$

(3) $$\begin{array}{c} \theta_{\mathrm{EM}}^{\left(n+1\right)}=\frac{\theta^{\left(n\right)}}{A^{T}1}A^{T}\left(\frac{m}{A\theta^{\left(n\right)}+b}\right) \end{array}$$

where *A* is the system matrix, ***b*** is the scatters and randoms (background counts), and ***q*** is the model of the expectation of the measured projection data. In addition to the measured PET data which are susceptible to noise, alternative sources of information can be incorporated into the reconstruction process to reduce the noise and PVEs in the reconstructed image. As alluded to above, the inclusion of prior information (in particular anatomical boundaries) can be achieved through either kernel (reparameterization) or MAP (regularisation) reconstruction methods. The particular methods investigated are summarized in Tables 1 and 2.

**Table 1:** Summary of the six magnetic resonance (MR)‐informed methods expressed in terms of the generalized Gaussian [Eq. (19)], indicating how each weight matrix or basis function is derived.

Method	$z_{j}\left(x,\theta^{\left(n\right)},r\right)$	MR‐derived weights	PET derived weights	*k*‐nearest neighbors	Weights and basis functions
Regularization					
Gaussian MR‐guided	$\frac{x_{j}}{\sigma_{\mathrm{MR}}}$	$e^{\frac{-{\left|\left|x_{j}-x_{l}\right|\right|}^{2}}{2\sigma_{\mathrm{MR}}^{2}}}$	1	Uses whole neighborhood	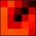
Bowsher	0	1	1	w.r.t. *x_j_*	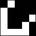
Anato‐functional	$\left[\frac{x_{j}}{\sigma_{\mathrm{MR}}},\frac{\theta_{j}^{\left(n\right)}}{\sigma_{\mathrm{PET}}}\right]$	$e^{\frac{-{\left|\left|x_{j}-x_{l}\right|\right|}^{2}}{2\sigma_{\mathrm{MR}}^{2}}}$	$e^{\frac{-{\left|\left|\theta_{j}^{\left(n\right)}-\theta_{l}^{\left(n\right)}\right|\right|}^{2}}{2\sigma_{\mathrm{PET}}^{2}}}$	Uses whole neighborhood	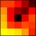
Reparameterization					
KEM	$\frac{x_{j}}{\sigma_{\mathrm{MR}}}$	$e^{\frac{-{\left|\left|x_{j}-x_{l}\right|\right|}^{2}}{2\sigma_{\mathrm{MR}}^{2}}}$	1	w.r.t. *x_j_*	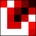
KEM LVS	$\left[\frac{x_{j}}{\sigma_{\mathrm{MR}}},\frac{r_{j}}{\sigma_{r}}\right]$	$e^{\frac{-{\left|\left|x_{j}-x_{l}\right|\right|}^{2}}{2\sigma_{\mathrm{MR}}^{2}}}e^{\frac{-{\left|\left|r_{j}-r_{l}\right|\right|}_{2}^{2}}{2\sigma_{\mathrm{MR}}^{2}}}$	1	w.r.t. $\left[\frac{x_{j}}{\sigma_{\mathrm{MR}}},\frac{r_{j}}{\sigma_{r}}\right]$	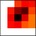
HKEM	$\left[\frac{x_{j}}{\sigma_{\mathrm{MR}}},\frac{\theta_{j}^{\left(n\right)}}{\sigma_{\mathrm{PET}}}\right]$	$e^{\frac{-{\left|\left|x_{j}-x_{l}\right|\right|}^{2}}{2\sigma_{\mathrm{MR}}^{2}}}$	$e^{\frac{-{\left|\left|\theta_{j}^{\left(n\right)}-\theta_{l}^{\left(n\right)}\right|\right|}^{2}}{2\sigma_{\mathrm{PET}}^{2}}}$	w.r.t. $\theta_{j}^{\left(n\right)}$	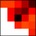

HKEM, hybrid kernel method; LVS, largest value sparsification; PET, positron emission tomography.

**Table 2:** Summary of each magnetic resonance (MR)‐informed method’s parameters. Each method underwent a restructured grid search, using a 10^8^ and 10^7^ count three‐dimensional‐simulated dataset, to determine the chosen parameters, which gave the best whole brain to positron emission tomography (PET)‐unique region structural similarity index (SSIM) trade‐off. The range of values over which the grid search was evaluated, and the chosen parameters are stated.

Method	Parameters for high‐count data			Parameters for low‐count data		
	Fixed	Varied	Chosen	Fixed	Varied	Chosen
Regularization						
Gaussian MR‐guided	N/A	$\sigma_{\mathrm{MR}}=0.01-5$ $\beta =0-10^{6}$	$\sigma_{\mathrm{MR}}=0.1$	N/A	$\sigma_{\mathrm{MR}}=0.01-5$ $\beta =0-10^{7}$	$\sigma_{\mathrm{MR}}=0.5$
Bowsher	N/A	k=0-60 $\beta =0-10^{6}$	k=20	N/A	k=0-60 $\beta =0-10^{7}$	k=40
Anato‐functional	$\sigma_{\mathrm{MR}}=0.1$	$\sigma_{\mathrm{PET}}=0.001-0.5$ $\beta =0-10^{6}$	$\sigma_{\mathrm{PET}}=0.01$	$\sigma_{\mathrm{MR}}=$ 0.5	$\sigma_{\mathrm{PET}}=0.001-0.5$ $\beta =0-10^{7}$	$\sigma_{\mathrm{PET}}=0.5$
Reparameterization						
KEM	N/A	$\sigma_{\mathrm{MR}}=0.01-5$ k=0-60	$\sigma_{\mathrm{MR}}=1$	N/A	$\sigma_{\mathrm{MR}}=0.01-5$ k=0-60	$\sigma_{\mathrm{MR}}=$ *0.5*
KEM LVS	$\sigma_{s}$= 40	$\sigma_{\mathrm{MR}}=0.01-5$ k=0-60	$\sigma_{\mathrm{MR}}=0.5$	$\sigma_{s}$= 40	$\sigma_{\mathrm{MR}}=0.01-5$ k=0-60	$\sigma_{\mathrm{MR}}=0.5$
HKEM	$\sigma_{\mathrm{MR}}=0.1$ $k_{\mathrm{MR}}=124$	$\sigma_{\mathrm{PET}}=0.001-0.5$ $k_{\mathrm{PET}}=0-124$	$\sigma_{\mathrm{PET}}=0.01$	$\sigma_{\mathrm{MR}}=0.5$,$k_{\mathrm{MR}}=124$	$\sigma_{\mathrm{PET}}=0.001-0.5$ $k_{\mathrm{PET}}=0-124$	$\sigma_{\mathrm{PET}}=0.5$

#### 2.1.1 Kernel method

MR‐informed KEM reparameterizes the emission image into a set of MR‐derived spatial basis functions (*K*).

(4) $$\begin{array}{c} \theta =K\alpha \end{array}$$

The coefficients $\left(\alpha \right)$ of these spatial basis functions (*K*) are estimated through maximizing the reparameterized Poisson log likelihood objective function, with respect to the basis function coefficients. Through the reparameterization of the emission image, and the nonnegativity constraint of the EM algorithm, the possible solution space is restricted. Provided the basis functions are of sufficient size, the emission image will be inhibited from forming noisy images, while retaining shared PET‐MR structures.

**Figure 1:**
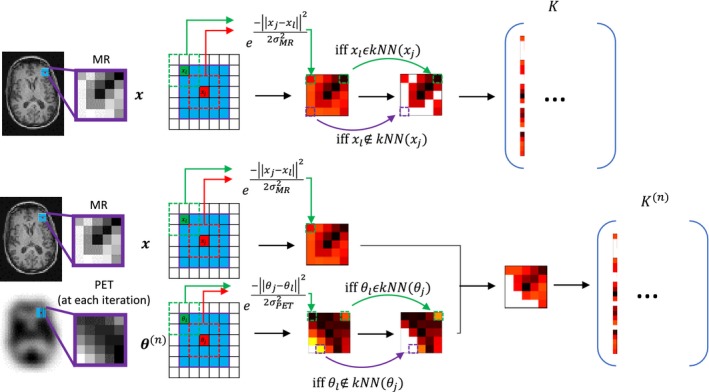
The formation of the kernel basis functions for the MR‐informed kernel (top) and the hybrid kernel (bottom). The neighborhood comparison is shown for the generalized patch case. The resulting basis functions are equivalent to the weighting factors used in the magnetic resonance (MR)‐guided Gaussian and anato‐functional maximum a posteriori methods, provided the number of *k*‐nearest neighbors is equal to the spatial neighborhood. [Color figure can be viewed at http://wileyonlinelibrary.com]

Kernel basis functions are spatial similarity matrices between each voxel and their neighboring voxels. Each kernel basis function is described through comparing a central voxel with its neighboring voxels. Only comparing voxels that lie within a predefined spatial neighborhood prevents the inclusion of long‐range correlations and reduces the computational burden. In a similar vein to the Bowsher prior, only the *k*‐nearest neighbors (*k*NN) in feature space are selected to contribute to a given voxel’s basis function. The Euclidean distance was used to determine the *k‐*nearest neighbors. The values of the kernel basis functions (within the spatial neighborhood) are generally calculated from the radial Gaussian kernel [see Eq. (5) below], acting on the feature vector representation of each voxel. Figure 1 illustrates this basis function calculation for a generalized patch feature vector. For all methods compared, the spatial neighborhood has a side length of five and the feature vector is implemented as a scalar, simply by using only the individual voxel intensity. For MR‐informed KEM this gives

(5) $$K_{\mathrm{jl}}=\left\{\begin{array}{cc} e^{\frac{-\|x_{j}-x_{l}{\|}^{2}}{2\sigma_{\mathrm{MR}}^{2}}}, & x_{l}\;\in \;k\text{NN}\;\text{of}\;x_{j}\\ 0,\;\;\; & \text{otherwise}\; \end{array}\right.$$

where ***x*** refers to the MR voxel intensity values and $\sigma_{\mathrm{MR}}$ is the standard deviation applied for the MR voxel‐based Gaussian. The resultant kernel basis functions together form the kernel matrix *K*, with which the emission image is reparameterized. The kernel matrix for all kernel implementations is row normalized in accordance with.^9^ Through reparameterizing the EM update equation, the spatial basis function coefficients $\left(\alpha \right)$ are found, which maximize the likelihood with respect to the measured projection data:

(6) $$\begin{array}{c} \alpha^{\left(n+1\right)}=\frac{\alpha^{\left(n\right)}}{K^{T}A^{T}1}K^{T}A^{T}\left(\frac{m}{AK\alpha^{\left(n\right)}+b}\right) \end{array}$$

An alternative means of sparsifying the kernel method has been proposed in Ref. 11, which uses a composite feature vector based on a weighted combination of the MR voxel values and their spatial location:

(7) $$K_{\mathrm{jl}}=\left\{\begin{array}{c} e^{\frac{-{\left|\left|z_{j}\left(x_{j},r_{j}\right)-z_{l}\left(x_{l},r_{l}\right)\right|\right|}_{2}^{2}}{2}},z_{l}\;\in \;k\text{NN}\;\mathrm{of}z_{j}\\ 0,\;\;\;\;\;\;\;\text{otherwise} \end{array}\right.$$

(8) $$\begin{array}{c} z_{j}\left(x_{j},r_{j}\right)=\left(\begin{array}{c} \frac{x_{j}}{\sigma_{\mathrm{MR}}}\\ \frac{r_{j}}{\sigma_{s}} \end{array}\right) \end{array}$$

where ***r*** *_j_* refers to the spatial location of voxel *j* and $\sigma_{s}$ refers to the spatial standard deviation. This method will be referred to as KEM LVS and has been shown to lead to more localized basis functions (Fig. S1), and thus better recovery of PET‐unique regions. A further extension of the kernel method is HKEM. HKEM includes the reconstructed PET image at each iteration into the kernel basis function value calculations, extending the Gaussian term to be comprised of a Gaussian applied to the current intensities of the reconstructed PET image, in product with a Gaussian applied to the intensities of the MR image. $\sigma_{\mathrm{PET}}$ refers to the standard deviation applied for the PET voxel‐based Gaussian:

(9) $$\begin{array}{c} K_{\mathrm{jl}}^{\left(n\right)}=\left\{\begin{array}{cc} e^{\frac{-{\left|\left|x_{j}-x_{l}\right|\right|}^{2}}{2\sigma_{\mathrm{MR}}^{2}}}e^{\frac{-{\left|\left|\theta_{j}^{\left(n\right)}-\theta_{l}^{\left(n\right)}\right|\right|}^{2}}{2\sigma_{\mathrm{PET}}^{2}}}, & \theta_{l}^{\left(n\right)}\;\in \;k\text{NN of}\;\theta_{j}^{\left(n\right)}\\ 0,\;\;\; & \text{otherwise}\;\; \end{array}\right. \end{array}$$

The HKEM implementation by Ref. 50, 51, uses all voxels within a spatial neighborhood to contribute to a basis function. An alternative implementation of HKEM would be to implement the *k*‐nearest neighbors search based on both PET and MR voxel values weighted by their standard deviation, in a similar manner to KEM LVS.

#### 2.1.2 Maximum *a posteriori* (MAP)

Regularization of the objective function, in accordance with MAP or PL is achieved through the inclusion of a prior term *R* to the objective function, as follows:

(10) $$\begin{array}{c} \Omega =L\left(q\left(\theta \right);m\right)-\beta R\left(\theta \right) \end{array}$$

Markov random fields are a common choice of prior that identify interactions between short‐range neighboring voxels (called cliques). For this case, $\left(R\left(\theta \right)\right)$ is equivalent to the Gibbs energy function and is purely influenced by relationships between voxels within the same clique. The quadratic function can be chosen as the inter‐voxel penalty function, which will suppress the differences between neighboring voxels, encouraging a smooth radiotracer reconstruction. A general expression for the quadratic prior function is given by

(11) $$R\left(\theta \right)=\sum_{j}\sum_{l\in N_{j}}w_{\mathrm{jl}}{\left(\theta_{j}-\theta_{l}\right)}^{2}$$

with the weighting factors *w_jl_* quantifying how similar we expect the neighboring voxel $\theta_{l}$ to be to the central voxel $\theta_{j}$ within a given spatial neighborhood $\left(N_{j}\right)$. A separable form of the MAP objective function for this weighted quadratic prior term [Eq (11)] is found through implementing De Pierro's decoupling rule,^57^ which employs a surrogate penalty term (using the principle of optimization transfer). The separable iterative update formula is explicitly presented in Ref. 32, 58 and repeated here for clarity:

(12) $$\begin{array}{c} \theta_{j}^{\left(n+1\right)}=\frac{2\theta_{j,EM}^{\left(n+1\right)}\sum_{i}A_{\mathrm{ij}}}{D_{j}^{\left(n\right)}+\sqrt{{\left(D_{j}^{\left(n\right)}\right)}^{2}+4C_{j}B_{j}^{\left(n\right)}}} \end{array}$$

(13) $$\begin{array}{c} D_{j}^{\left(n\right)}=\sum_{i}A_{\mathrm{ij}}-\frac{\beta }{2}\sum_{l\in N_{j}}w_{\mathrm{jl}}\left(\theta_{j}^{\left(n\right)}+\theta_{l}^{\left(n\right)}\right) \end{array}$$

(14) $$\begin{array}{c} B_{j}^{\left(n\right)}=\theta_{j,EM}^{\left(n+1\right)}\sum_{i}A_{\mathrm{ij}} \end{array}$$

(15) $$\begin{array}{c} C_{j}=\beta \sum_{l\in N_{j}}w_{\mathrm{jl}} \end{array}$$

In the case of no anatomical information, the weighting factors are indiscriminately set to 1/*N_j_*, resulting in smoothing across uniform and boundary regions alike. Alternatively, the weighting factors can be obtained from a co‐registered MR image that shares boundary information with the PET data, to prevent over‐smoothing across shared PET‐MR boundaries. These weighting factors calculate the similarity between a chosen voxel and the voxels that lie within its spatial neighborhood. Two popular MR‐derived weights use the Gaussian and asymmetric Bowsher‐based formulations. Each method compares the central voxel's feature vector with the feature vector of each of its neighboring voxels, in turn. The Gaussian MR‐guided prior uses the radial Gaussian in feature space to determine the similarity of neighboring voxels, to a chosen voxel (again, as previously indicated, only single voxel scalar values are used as feature vectors in this work):

(16) $$\begin{array}{c} w_{\mathrm{jl}}=e^{\frac{-{\left|\left|x_{j}-x_{l}\right|\right|}^{2}}{2\sigma_{\mathrm{MR}}^{2}}} \end{array}$$

The weighting factors for all MAP methods are normalized, with the sum of all weighting factors within a given voxel's neighborhood set to 1. The Bowsher prior sets the weighting factors for the* k* most similar voxels (determined by the Euclidean distance in feature space) within a spatial neighborhood to 1, and the others to 0:

(17) $$\begin{array}{c} w_{\mathrm{jl}}=\left\{\begin{array}{cc} 1, & x_{l}\in k\text{NN}\;ofx_{j}\\ 0, & \text{otherwise}\;\; \end{array}\right. \end{array}$$

The anato‐functional reconstruction methodology extends the MR‐guided Gaussian prior to include similarity weights from the current PET image update. The composite weighting factor is the product between the PET and MR Gaussian similarity kernels, where the PET similarity kernel is calculated from the image for each iteration update:

(18) $$\begin{array}{c} w_{\mathrm{jl}}^{\left(n\right)}=e^{\frac{-{\left|\left|x_{j}-x_{l}\right|\right|}^{2}}{2\sigma_{\mathrm{MR}}^{2}}}e^{\frac{-{\left|\left|\theta_{j}^{\left(n\right)}-\theta_{l}^{\left(n\right)}\right|\right|}^{2}}{2\sigma_{\mathrm{PET}}^{2}}} \end{array}$$

As the anato‐functional and HKEM priors rely on a previous activity estimate, no theoretical convergence guarantee can be provided for these adaptively weighted regularization methods. Although, our preliminary findings showed that empirically these reconstruction methods converge to a fixed point solution at which the objective function remains unchanged. All methods presented in both the kernel and MAP sections are based on the formation of similarity weights between voxels confined within a spatial neighborhood. These weights, or kernel basis function values, can be described through the general expression [Eq. (19)], based on a composite feature vector (***z***). Subsequently, the* k* most similar voxels to the central voxel (the *k*‐nearest neighbors) are identified, where the voxels' similarity can be assessed in terms of single voxel intensity, patch intensity, spatial position, or any alternative feature vector. Weighting factors (or basis function elements) that correspond to voxels that lie outside the central voxel’s *k*‐nearest neighbors are set to zero. All MR‐informed methods are expressed in this formulation in Table 1.

(19) $$\begin{array}{c} w_{\mathrm{jl}}^{\left(n\right)}\;\text{or}\;K_{\mathrm{jl}}^{\left(n\right)}e^{\frac{-\|z_{j}\left({x,\theta }^{\left(n\right)},r\right)-z_{l}\left(x,\theta^{\left(n\right)},r\right){\|}_{2}^{2}}{2}} \end{array}$$

### 2.2 Simulation studies

**Figure 2:**
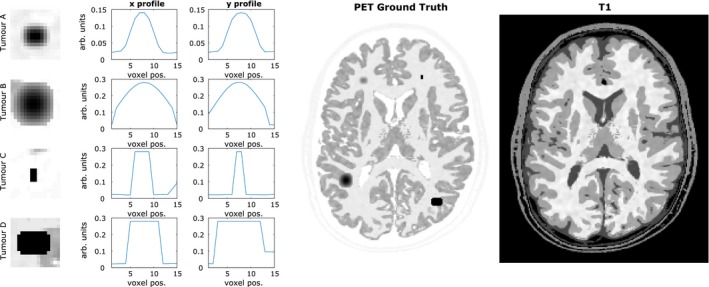
(Left) Simulated positron emission tomography (PET)‐unique high‐intensity regions (tumors A, B, C and D) that differ in structure to the corresponding regions in the T1 magnetic resonance (MR) phantom. (center) Simulated [^18^F]fluorodeoxyglucose (FDG) PET phantom based on the BrainWeb phantom, with the four PET‐unique high‐intensity regions added. (right) T1 image. All images are shown at MR resolution. The gray matter intensity value shown in the tumor profiles is approximately 0.1 arbitrary units. [Color figure can be viewed at http://wileyonlinelibrary.com]

A three‐dimensional‐simulated [^18^F]fluorodeoxyglucose (FDG) PET phantom was constructed with a voxel‐side length of 1 mm, based on the BrainWeb^59^ segmented MR database. The gray and white matter tissue classes were assigned intensities with the ratio of 4:1 in keeping with the expected uptake from an FDG tracer.^30^, ^60^, ^61^ Real PET images have more structural variation than the produced piecewise constant simulated phantom. To discourage overly piecewise constant images, Gaussian smoothed random structures were incorporated into the simulated PET phantom, in accordance with Eq. (20), producing more varied tissue structure:

(20) $$\begin{array}{c} \theta_{\mathrm{rand}}=\theta \left(1+H\left(2G\left(rand,\sigma_{\mathrm{sm}}\right)-1\right)\right) \end{array}$$

where *rand* are uniformly distributed random numbers between 0 and 1 for each image voxel, *G* corresponds to convolution with a Gaussian kernel of width $\sigma_{\mathrm{sm}}$, and *H* is an amplitude parameter. Four high‐intensity structures (lesions) were added to the PET phantom (only). The two smaller lesions are located in the white matter, whereas the larger lesions are positioned across MR boundaries that differ (Fig. 2). Profiles through each of these PET‐unique regions are also shown in Fig. 2, with the intensity of tumors C and D equal to three times the intensity of the gray matter. The PET phantom was projected into span 11 sinograms using a reconstruction software which models the Siemens Biograph mMR PET‐MR scanner.^62^ A 4.5 mm point spread function (PSF) was applied in the forward model, to simulate the overall effects of photon acollinearity, positron range, and finite crystal width.^63^ The simulated sinograms were rescaled to high‐ and low‐count level datasets (10^8^ and 10^7^ prompts, respectively), each with a randoms fraction of 20% and a scatter fraction of 20%, prior to introducing Poisson noise. This was repeated for 10 noise realizations. The simulated sinograms were reconstructed at mMR resolution (voxel size of 2.09 × 2.09 × 2.03 mm^3^), with resolution modeling (PSF) included in the reconstruction.

#### 2.2.1 Simulation studies parameter selection

All methods underwent a restricted grid search of the parameter range, for both the high‐count and low‐count simulated datasets. All images were reconstructed up to 300 iterations, with images resized to the original 1 mm resolution of the ground truth phantom for the calculation of the error metrics. The structural similarity index (SSIM)^64^ was used to determine the similarity between the reconstructed images and the ground truth, as is generally perceived to provide a closer numerical representation of visual perception. SSIM has previously been used in the PET and image processing literature to evaluate novel methodologies,^54^, ^65^, ^66^ and to assess structural similarity between multimodality images as part of a proposed methodology,^17^ among other uses. The simplified formulation of SSIM employed in this work is

(21) $$\begin{array}{c} SSIM\left(\theta ,\theta^{\mathrm{GT}}\right)=\frac{\left(2\mu_{\theta^{\mathrm{GT}}}\mu_{\theta }+C_{1}\right)\left(2\sigma_{\theta^{\mathrm{GT}}\theta }+C_{2}\right)}{\left(\mu_{\theta }^{2}+\mu_{\theta^{\mathrm{GT}}}^{2}+C_{1}\right)\left(\sigma_{\theta }^{2}+\sigma_{\theta^{\mathrm{GT}}}^{2}+C_{2}\right)} \end{array}$$

where $\mu_{\theta }$ refers to the spatial local mean, $\sigma_{\theta }$ refers to the local standard deviation, and $\sigma_{\theta^{\mathrm{GT}}\theta }$ is the cross‐covariance between the image $\theta^{\mathrm{GT}}$ and θ. *C*_1_ and *C*_2_ are constant values dependent on the dynamic range of the image. The parameters for each reconstruction method were compared for their ability to improve the SSIM metric, for regions where the PET and MR phantom notably differ (PET‐unique regions), and for the whole brain region. For each reconstruction method, the SSIM trade‐off curve that is positioned closest to the SSIM value of 1 for both the whole brain and PET‐unique regions, that is, produced the largest sum of the unique SSIM squared and whole brain SSIM squared, was selected. The chosen parameters for each reconstruction method are presented in Table 2. It should be noted that the performance of the PET‐MR‐informed methods of HKEM, and anato‐functional was found to be highly sensitive to the chosen parameters (in particular $\sigma_{\mathrm{PET}}$) and the noise level of the PET image under reconstruction.

#### 2.2.2 Simulation studies multiple noise realizations

**Figure 3:**
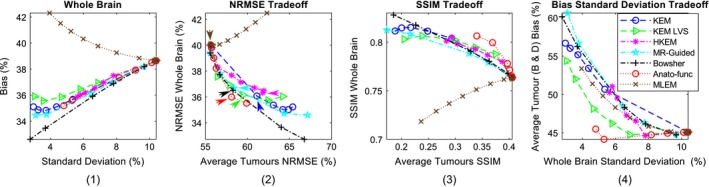
All the results shown are for the high counts (10^8^) simulated dataset, over multiple noise realizations. (1) Bias vs standard deviation for the whole brain region. (2) Whole brain normalized root mean square error (NRMSE) vs the positron emission tomography (PET)‐unique region NRMSE (averaged over the four PET‐unique regions). (3) Whole brain structural similarity index (SSIM) vs the PET‐unique region SSIM (averaged over the four PET‐unique regions). (4) PET‐unique region bias (averaged over tumor regions B and D only) vs whole brain standard deviation. The methods investigated are shown for increasing β values (MAP) or *k*‐nearest neighbors (KEM). Maximum likelihood expectation maximization is shown for increasing levels of post reconstruction smoothing. [Color figure can be viewed at http://wileyonlinelibrary.com]

The chosen parameter ranges for each reconstruction method were employed to reconstruct 10 noise realizations of the simulated data, for both the 10^8^ and 10^7^ count levels. The reconstructed images were assessed using a variety of error and image quality metrics for individual voxels and across regions of interest (ROI), to determine which MR‐informed method provided the best trade‐off between the reconstruction of the whole brain and PET‐unique regions. The values calculated for SSIM (Figs. 3 and 6) are now for multinoise realization data and were averaged across all noise realizations. The voxel‐wise metrics of mean, bias, and standard deviation (presented in the Appendix for tumor regions Figs. S2 and S3) are calculated across the multiple noise realizations (*N_noise_*) in accordance with the following equations:

(22) $$\begin{array}{c} {\bar{\theta }}_{j}=\frac{1}{N_{\mathrm{noise}}}\sum_{n}^{N_{\mathrm{noise}}}\theta_{j,n} \end{array}$$

(23) $$\begin{array}{c} Bias_{j}={\bar{\theta }}_{j}-\theta_{j}^{\mathrm{GT}} \end{array}$$

(24) $$\begin{array}{c} \sigma_{j}=\sqrt{\frac{1}{N_{\mathrm{noise}}}\sum_{n}^{N_{\mathrm{noise}}}{\left(\theta_{j,n}-{\bar{\theta }}_{j}\right)}^{2}} \end{array}$$

ROI‐based error metrics of bias and standard deviation are also calculated across multiple noise realizations. These metrics are an extension of the metrics above; the metric value (squared) is now summed over each voxel within the ROI and is normalized according to the ground truth. ROI bias vs standard deviation curves are presented for the whole brain region, and each of the PET‐unique regions (Figs. 3, 6; Figs. S2 and S3).

(25) $$\begin{array}{c} Bias_{\mathrm{ROI}}\left(\%\right)=\sqrt{\frac{\sum_{j}^{N_{\mathrm{ROI}}}{\left({\bar{\theta }}_{j}-\theta_{j}^{\mathrm{GT}}\right)}^{2}}{\sum_{j}^{N_{\mathrm{ROI}}}{\left(\theta_{j}^{\mathrm{GT}}\right)}^{2}}}\times 100 \end{array}$$

(26) $$\begin{array}{c} \sigma_{ROI,1}\left(\%\right)=\sqrt{\frac{\sum_{n}^{N_{\mathrm{noise}}}\sum_{j}^{N_{\mathrm{ROI}}}{\left({\bar{\theta }}_{j}-\theta_{j,n}\right)}^{2}}{N_{\mathrm{noise}}\sum_{j}^{N_{\mathrm{ROI}}}{\left(\theta_{j}^{\mathrm{GT}}\right)}^{2}}}\times 100 \end{array}$$

Normalized root mean square error (NRMSE) as shown in Figs. 3 and 6, over a specific ROI can then be calculated as:

(27) $$NRMSE\left(\%\right)=\sqrt{Bias_{\mathrm{ROI}}^{2}+\sigma_{ROI,1}^{2}}=\sqrt{\frac{\sum_{n}^{N_{\mathrm{noise}}}\sum_{j}^{N_{\mathrm{Roi}}}{\left(\theta_{j,n}-\theta_{j}^{\mathrm{GT}}\right)}^{2}}{N_{\mathrm{Noise}}\sum_{k}{\left(\theta_{k}^{\mathrm{GT}}\right)}^{2}}}\times 100$$

Alternative error metrics that only depend on a single noise realization are also included to allow direct comparison with the real data studies (Figs. 5, 8, 10 and 12). The tumor mean values were compared to the white matter standard deviation (calculated using an eroded ROI) to allow the trade‐off between the retention of PET‐unique features and noise suppression of each method to be assessed. These single noise realization error metrics are

(28) $$\begin{array}{c} {\bar{\theta }}_{\mathrm{ROI}}=\frac{1}{N_{\mathrm{ROI}}}\sum_{j}^{N_{\mathrm{ROI}}}\theta_{j} \end{array}$$

(29) $$\begin{array}{c} \sigma_{\text{ROI},2}=\sqrt{\frac{1}{N_{\mathrm{ROI}}}\sum_{j}^{N_{\mathrm{ROI}}}{\left(\theta_{j}-{\bar{\theta }}_{\mathrm{ROI}}\right)}^{2}} \end{array}$$

However the tumor mean vs white matter standard deviation metric is incomplete and does not penalize the distortion of tumor regions, bias of the cortical regions, or the smoothing of different intensities across regional boundaries (PVEs). To address the issue of PVEs, the mean value of the white matter, gray matter, and cerebral spinal fluid (CSF) regions (using the full noneroded ROI including edges) is also examined. These regional mean values are plotted against their corresponding standard deviation values. The regional standard deviation values used for these plots are also calculated using the full noneroded ROI. Methods that reduce PVEs across these regional boundaries should result in a reduction in white matter and CSF mean values, and an increase in the gray matter mean value relative to unsmoothed MLEM, (while over‐smoothing methods will produce the converse trends). Improved reconstruction methods should therefore ideally lead to a fixed tumor mean relative to unsmoothed MLEM, reduced white matter standard deviation relative to unsmoothed MLEM, and follow the aforementioned regional mean trends.

### 2.3 Real data studies

#### 2.3.1 FDG dataset

Real [^18^F]FDG data from a patient scan (Alzheimer's disease) was reconstructed using all seven methods previously considered. The dataset was acquired from the Siemens Biograph mMR simultaneous PET‐MR scanner, allowing simple acquisition of co‐registered PET‐MR data. The [^18^F]FDG scan had a total prompt count of 4.69 × 10^8^ and a scan duration of 23 min. The tracer activity at time of injection (81 minutes prior to start of image acquisition) was 229 MBq. A T1 MPRAGE scan provided the anatomical image, which was resampled to the PET resolution (2.08626 mm × 2.08626 mm × 2.03125 mm) for use in anatomical guidance.

#### 2.3.2 Augmented FDG dataset

Four simulated tumors were added to the FDG dataset to give known PET‐unique regions relative to the corresponding MR structure. The intensity profiles of the tumor regions are shown in Fig. 9 (the PET intensity of the gray matter is approximately 0.4 arb. units). This augmented dataset is reconstructed at the normal full (100%) count level and then also resampled to a lower count level of 10% (relative to the total prompt count level). The parameters used for the 100% and 10% count level‐augmented FDG real data studies were those chosen for the high‐ and low‐count simulation studies, respectively. However, the $\sigma_{\mathrm{PET}}$ parameters employed by the anato‐functional method and HKEM were reselected for each count level, due to the dependence of $\sigma_{\mathrm{PET}}$ on the PET image intensity. As previously evaluated in the simulated data studies, the mean value of each PET‐unique region was compared to the standard deviation of an eroded white matter region, in order to assess the ability of each method to simultaneously recover the PET‐unique region while suppressing noise in an (assumed) approximately uniform region. The white matter mask was extracted from the T1 image using FSL.^67^ The mean value of the white matter, gray matter, and cerebral spinal fluid (CSF) regions (using the full noneroded ROI including edges) is also examined for the real data studies, to help in investigating the PVE properties of each reconstruction method.

## 3 Results

### 3.1 Simulation Studies

For most of the figures presented (Figs. 3, 5, 6, 8; Figs. S2 and S3), either the regularization parameter $\left(\beta \right)$ is increased (for MAP methods), or the number of *k*‐nearest neighbors is increased (for kernel methods) along each curve. The original full neighborhood implementation of HKEM^50^, ^51^ is equivalent to the last point on each HKEM curve (*k_PET_ *= 124). The popular reconstruction method of MLEM is also shown for comparison, at varying levels of postreconstruction smoothing.

#### 3.1.1 Simulation Studies High Counts

Figure 3 shows a series of trade‐off curves for all reconstruction methods under investigation, applied to the 10 noise realizations of the high‐count (10^8^) simulated dataset. The bias vs standard deviation plot for the whole brain region (Fig. 3 column 1) demonstrates the improvements of all regularized and reparameterized MR‐informed and PET‐MR‐informed reconstruction methods over postsmoothed MLEM, across the whole brain region where the majority of the MR structure matches the PET. The use of MR information in these methods enables smoothing across MR uniform regions (reducing noise), while preserving MR boundaries and reducing PVEs. Of particular interest in this work is the ability to maintain these positive attributes of MR‐informed methods, while also maintaining genuine high‐intensity features (tumor regions) present only in the PET data and not in the MR. To address this question, both NRMSE and SSIM curves are presented in Fig. 3 (columns 2 and 3 respectively), showing the trade‐off between the accurate reconstruction of the whole brain region vs the PET‐unique regions (averaged over the four PET‐unique regions). Both the NRMSE and SSIM curves (Fig. 3) show that the anato‐functional method achieves the best trade‐off relative to the other investigated methods, although the improvement is less apparent in terms of NRMSE. The KEM LVS and HKEM also perform well in terms of the SSIM trade‐off between the PET‐unique and whole brain regions, whereas if evaluated in terms of the NRMSE trade‐off, the Bowsher method performs more favorably. The PET‐unique region bias vs whole brain region standard deviation trade‐off curve (Fig. 3 column 4) also demonstrates the improved performance of the anato‐functional method and KEM LVS, both attaining a reduced bias (for the PET‐unique regions B & D) for a fixed whole brain standard deviation (image noise level) in comparison with postsmoothed MLEM.

**Figure 4:**
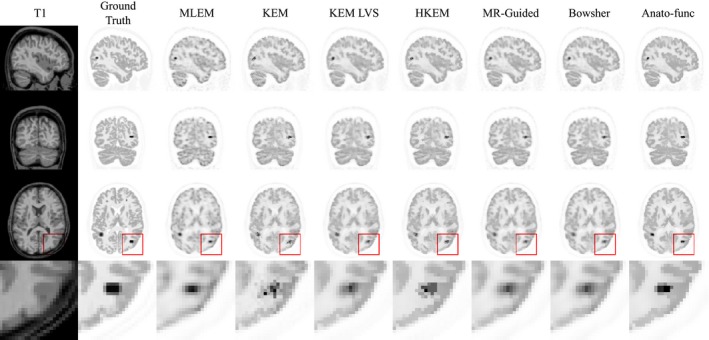
All the results shown are for the high counts (10^8^) simulated dataset for a single noise realization, at positron emission tomography (PET) resolution. Reconstructed images for each of the methods under investigation. A zoomed in transverse region is shown along the bottom row. All magnetic resonance (MR)‐informed methods are shown at approximately fixed whole brain normalized root mean square error (NRMSE, as indicated by the arrows on the NRMSE curves, Fig. 3 column 2. [Color figure can be viewed at http://wileyonlinelibrary.com]

The points indicated by arrows on the NRMSE curves (Fig. 3 column 2) indicate the parameters that produce approximately equivalent whole brain NRMSE. The corresponding images with fixed whole brain NRMSE are shown in Fig. 4, in which all MR‐informed methods can be seen to yield a sharper reconstruction of the regions with shared PET‐MR structure, in comparison with postsmoothed MLEM. Additionally, the anato‐functional method can be seen to perform favorably in reconstructing PET‐unique regions with a relatively high, constant intensity value and well‐defined boundaries (e.g., tumor D, Fig. 4 bottom row), whereas the MR‐guided and Bowsher MAP methods and KEM LVS perform better in the reconstruction of high‐intensity PET‐unique regions with a smoothed structure (e.g., tumor B).

**Figure 5:**
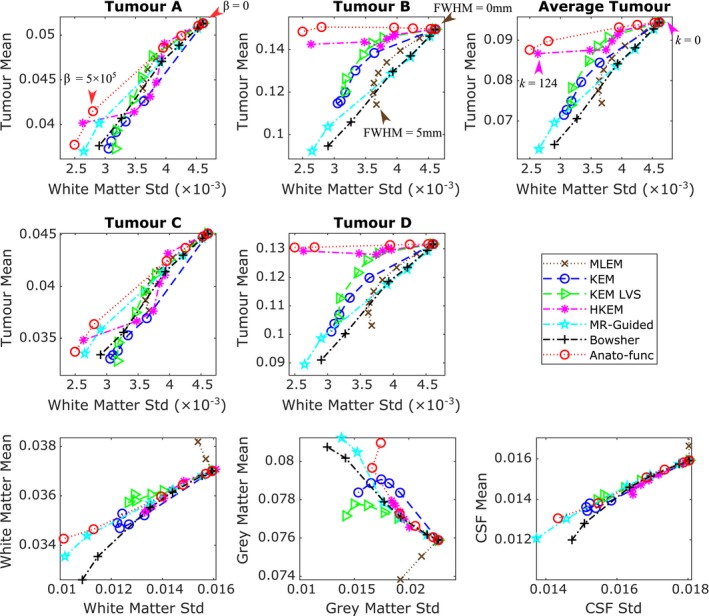
All the results shown are for the high counts (10^8^) simulated dataset, averaged over the multiple noise realizations. (top and middle row) The tumor mean value vs white matter standard deviation, for all methods under consideration, for each positron emission tomography (PET)‐unique region. The mean and standard deviation values were calculated within the specified (eroded) regions of interest (ROIs) at PET resolution, and then averaged across the multiple noise realizations. The maximum likelihood expectation maximization (MLEM) noise‐free mean values for the different tumor regions are: 0.0624 for tumor A, 0.155 for tumor B, 0.0575 for tumor C and 0.137 for tumor D. (bottom row) Regional mean value vs regional standard deviation using the full ROI including edges. The expected trend for methods that reduce partial volume effects is a reduction in the white matter and cerebral spinal fluid mean, and an increase in the gray matter mean. The *β* (MAP) or *k* (KEM) parameter values are increased along each curve. MLEM is also shown for increasing level of postreconstruction smoothing. [Color figure can be viewed at http://wileyonlinelibrary.com]

The ability of each reconstruction method to suppress image noise, without in turn suppressing the PET‐unique regions was also assessed through tumor mean vs white matter standard deviation curves (Fig. 5). These graphs show the improvement attained by the anato‐functional method, HKEM and to lesser extent, KEM LVS relative to the other MR(only)‐informed reconstruction methodologies for the high‐intensity tumor regions B and D. The anato‐functional method and HKEM can achieve mean values similar to that of unsmoothed MLEM for the high‐intensity PET‐unique regions, while markedly reducing the standard deviation across the white matter region. All MR‐informed and PET‐MR‐informed methods also lead to a reduction in the white matter mean and an increase in the gray matter mean in comparison with MLEM (Fig. 5, bottom row), as expected for methods that reduce the PVEs in these regions. This demonstrates the capability of MR‐informed methods to counteract PVEs that cause spill‐out effects for high‐intensity regions. The PET‐unique regions of A and C appear to have insufficient intensity to really benefit from the inclusion of PET information in the PET‐MR‐informed methods, with both the MR‐informed and PET‐MR‐informed methods producing similar tumor mean vs white matter standard deviation curves (Fig. 5, top and middle rows), as such, the information that can be drawn from the PET‐unique regions A and C is limited.

#### 3.1.2 Simulation studies low counts

**Figure 6:**
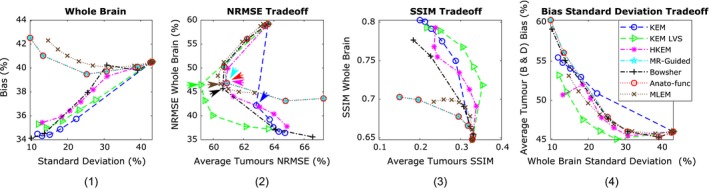
All the results shown are for the low counts (10^7^) simulated dataset, using multiple noise realizations. (1) Bias vs standard deviation for the whole brain region. (2) Whole brain normalized root mean square error (NRMSE) vs the positron emission tomography (PET)‐unique region NRMSE (averaged over the four PET‐unique regions). (3) Whole brain structural similarity index (SSIM) vs the PET‐unique region SSIM (averaged over the four PET‐unique regions). (4) PET‐unique region bias (averaged over tumor regions B and D only) vs whole brain standard deviation. Each method is shown for increasing *β* values (MAP) or *k*‐nearest neighbors (KEM). Maximum likelihood expectation maximization is shown for increasing levels of post reconstruction smoothing. [Color figure can be viewed at http://wileyonlinelibrary.com]

Figure 6 (columns 2 and 3) shows the NRMSE and SSIM trade‐off curves between the PET‐unique (averaged over the four PET‐unique regions) and whole brain regions, for the low count (10^7^) simulated dataset. Both the NRMSE and SSIM plots show KEM LVS performing better than the other reconstruction methods, achieving both an improvement in whole brain and PET‐unique region image quality in comparison with unsmoothed MLEM. This trend can also be clearly seen in the bias (for tumor regions B and D) vs whole brain standard deviation plot (Fig. 6 column 4), with KEM LVS achieving the lowest tumor bias for a fixed whole brain noise level. The Bowsher method also performs well, when evaluated in terms of NRMSE trade‐off (Fig. 6 column 2), but less so if evaluated in terms of SSIM trade‐off (Fig. 6, column 3) or bias vs standard deviation trade‐off (Fig. 6, column 4) where instead the HKEM performs better than the other methods excluding KEM LVS.

**Figure 7:**
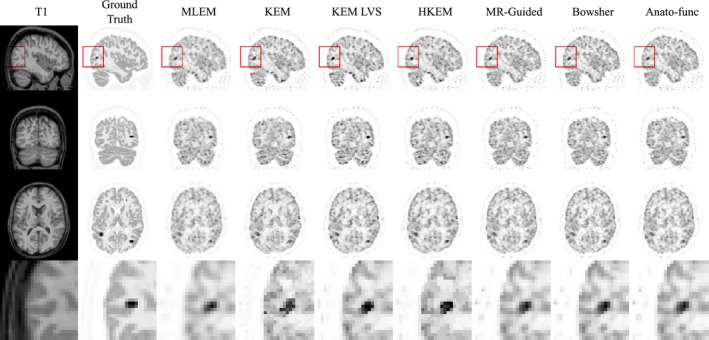
All the results shown are for the low counts (10^7^) simulated dataset, for single noise realization, at positron emission tomography (PET) resolution. Reconstructed images for each of the methods under investigation. A zoomed in sagittal region is shown along the bottom row. All magnetic resonance‐informed methods are shown at approximately fixed whole brain normalized root mean square error (NRMSE), as indicated by arrows on the NRMSE curves, Fig. 6 column 2. MLEM is shown with post reconstruction smoothing with a FWHM of 3.5 mm. [Color figure can be viewed at http://wileyonlinelibrary.com]

**Figure 8:**
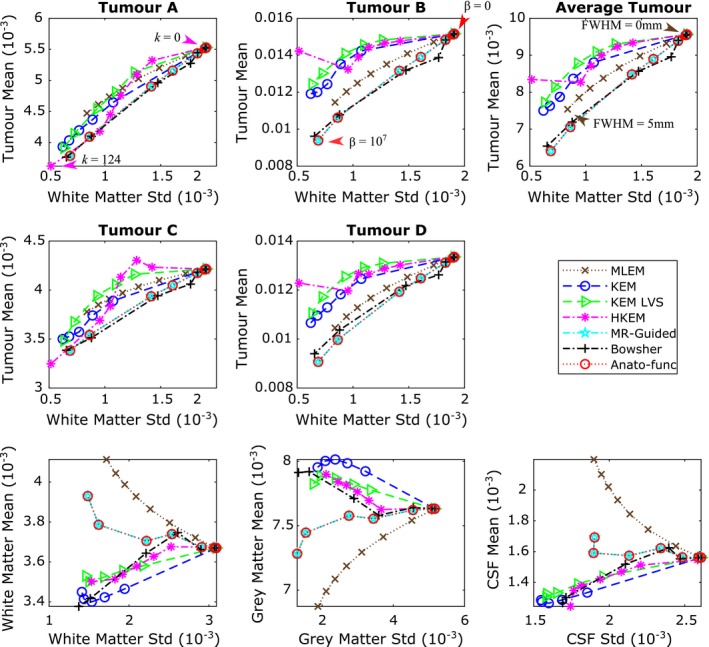
All the results shown are for the low counts (10^7^) simulated dataset, averaged over multiple noise realizations. (top and middle row) The tumor mean value vs white matter standard deviation, for all methods under consideration, for each positron emission tomography (PET)‐unique region. The mean and standard deviation values were calculated within the specified regions of interests (ROIs) at PET resolution, and then averaged across the multiple noise realizations. The maximum likelihood expectation maximization (MLEM) noise‐free mean values for the different tumor regions are: 0.00624 for tumor A, 0.0155 for tumor B, 0.00575 for tumor C and 0.0137 for tumor D. (bottom row) Regional mean value vs regional standard deviation using the full ROI including edges. The expected trend for methods that reduce partial volume effects is a reduction in the white matter and cerebral spinal fluid mean, and an increase in the gray matter mean. *β* (MAP) or *k* (KEM) parameter values were increased along each curve. MLEM is also shown for increasing level of postreconstruction smoothing. [Color figure can be viewed at http://wileyonlinelibrary.com]

The points indicated by arrows on the NRMSE curves (Fig. 6, column 2) correspond to the parameters that produce approximately equivalent whole brain NRMSE, with the reconstructed images shown in Fig. 7. In Fig. 7, KEM LVS and HKEM show a marginally sharper recovery of the PET‐unique regions in comparison with the remaining methods. Figure 8 shows the tumor mean vs white matter region standard deviation for the low count dataset, with KEM LVS and HKEM performing well in comparison to the other methods investigated. The two high‐intensity PET‐unique regions (B and D), particularly demonstrate the noise suppression abilities of the KEM LVS and HKEM methods, for a fixed tumor mean value. KEM also achieves a reduced white matter standard deviation for a relatively fixed PET‐unique region mean value in Fig. 8, despite visually its PET‐unique regions being more distorted than the corresponding KEM LVS and HKEM PET‐unique regions (as shown in Fig. 7). This highlights the limitations of using ROI‐based error metrics. Figure 8 (bottom row) shows an increase in the white matter mean value and a reduction in the gray matter mean value for the MR‐guided and anato‐functional methods due to an increased smoothing across the white–gray matter boundary (PVEs). Therefore, the MR‐guided and anato‐functional methods (with their low count chosen parameters) have lost their PVE correction capabilities that were demonstrated in the high counts simulation studies (Fig. 5).

### 3.2 Real data studies

#### 3.2.1 100% count level‐augmented FDG dataset

**Figure 9:**
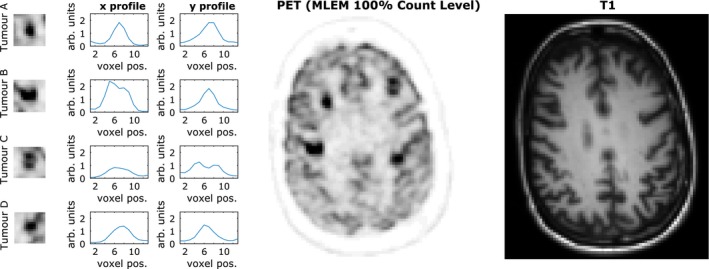
Real patient [^18^F]fluorodeoxyglucose (FDG) dataset, augmented with simulated tumor regions, to produce genuine regions of positron emission tomography (PET)‐magnetic resonance (MR) mismatch. (left) Tumor regions and profiles from the maximum likelihood expectation maximization (MLEM) reconstructed image for the 100% count level. (middle) MLEM reconstructed image of the whole brain region, shown at 300 iterations for the 100% count level. (right) T1 MPRAGE image. All images shown at PET resolution. The gray matter PET intensity value is approximately 0.4 arb. Units. [Color figure can be viewed at http://wileyonlinelibrary.com]

**Figure 10:**
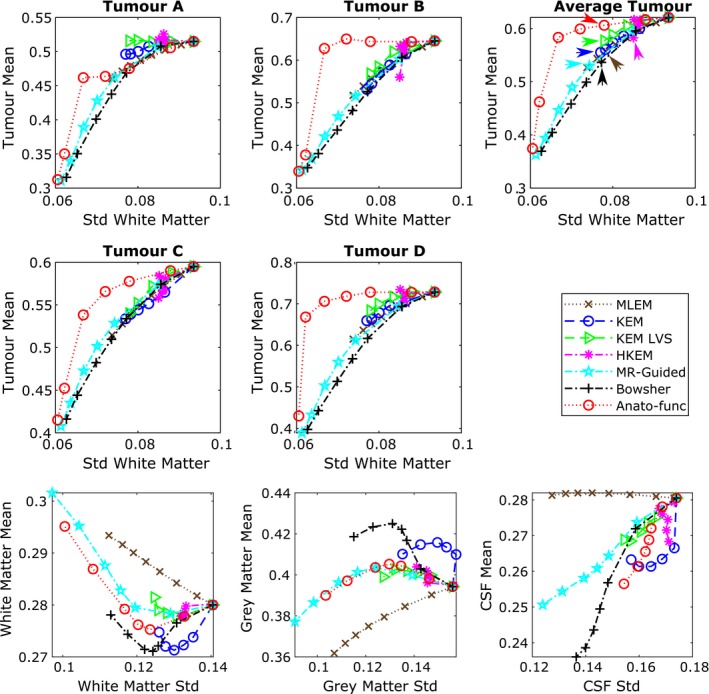
All results shown are for the 100% count level‐augmented real dataset. (top and middle rows) Tumor mean vs white matter (eroded mask) standard deviation. The arrows indicate the mean vs standard deviation trade‐off of the chosen parameters for the images shown in Fig. 11. (bottom row) Regional mean value vs regional standard deviation using the full regions of interest including edges. The expected trend for methods that reduce partial volume effects is a reduction in the white matter and cerebral spinal fluid mean, and an increase in the gray matter mean. [Color figure can be viewed at http://wileyonlinelibrary.com]

**Figure 11:**
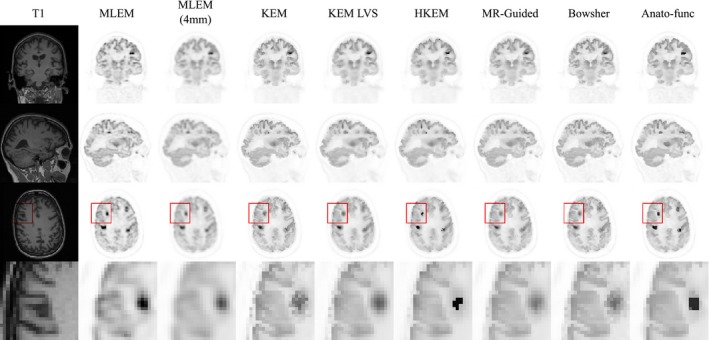
The reconstructed images are shown for each of the reconstruction methods investigated, applied to the 100% count level‐augmented real dataset. A zoomed in transverse region is shown along the bottom row. All magnetic resonance‐informed methods are shown at approximately fixed white matter standard deviation, as indicated by the arrows in Fig. 10. Maximum likelihood expectation maximization is shown with postreconstruction smoothing (FWHM 4 mm). [Color figure can be viewed at http://wileyonlinelibrary.com]

Figure 10 shows the tumor mean vs white matter standard deviation trade‐off plots (top and middle row) in addition to the white matter, gray matter, and CSF regional mean plots (bottom row), for the augmented FDG dataset (Fig. 9), at the 100% count level. The anato‐functional method is shown to perform well for the high‐count real data studies, achieving the lowest white matter standard deviation (noise level) for a fixed tumor mean value (for three out of the four tumor regions). The KEM LVS and HKEM methods also perform better than the remaining MR‐informed methods when evaluated in terms of tumor mean vs white matter standard deviation trade‐off (Fig. 10). Figure 10 (bottom row) shows that all MR‐informed methods produce images with a reduced white matter mean, and an increased gray matter mean for moderate levels of regularization (or reparameterization). These trends correspond to a reduction in PVEs, in particular for the KEM and Bowsher methods. Figure 11 shows the reconstructed images for each of the MR‐informed methods, with the parameters chosen to present an approximately fixed white matter standard deviation (as indicated by the arrowheads in Fig. 10). Figure 11 also shows the improved definition and delineation between the white and gray matter when using the Bowsher method and KEM.

#### 3.2.2 10% count level‐augmented FDG dataset

**Figure 12:**
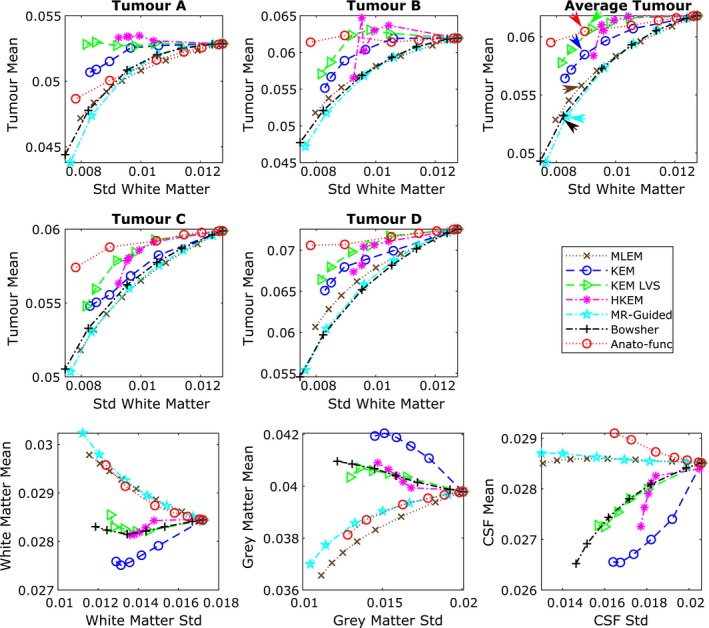
All results shown are for the 10% count level‐augmented real dataset. (top and middle row) Tumor mean vs white matter (eroded mask) standard deviation. The arrows indicate the mean vs standard deviation trade‐off of the chosen parameters for the images shown in Fig. 13. (bottom row) Regional mean value vs regional standard deviation using the full regions of interest including edges. The expected trend for methods that reduce partial volume effects is a reduction in the white matter and cerebral spinal fluid mean, and an increase in the gray matter mean. [Color figure can be viewed at http://wileyonlinelibrary.com]

**Figure 13:**
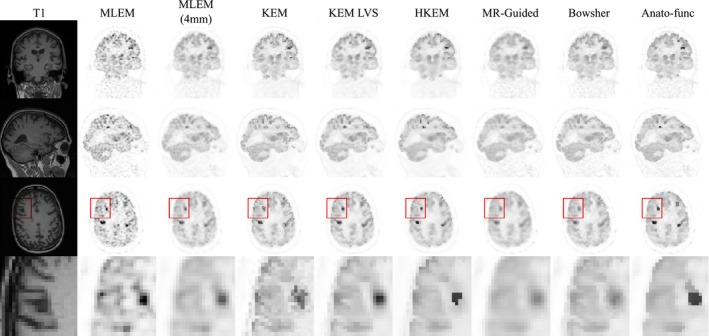
The reconstructed images are shown for each of the reconstruction methods investigated, applied to the 10% count level‐augmented real dataset. A zoomed in transverse region is shown along the bottom row. All magnetic resonance‐informed methods are shown at approximately fixed white matter standard deviation, as indicated by arrows in, Fig. 12. Maximum likelihood expectation maximization is shown with postreconstruction smoothing (FWHM 4 mm). [Color figure can be viewed at http://wileyonlinelibrary.com]

Figure 12 shows the tumor mean values vs white matter standard deviation plots (top and middle row) in addition to the white matter, gray matter, and CSF regional mean plots (bottom row), for the augmented FDG dataset at the 10% count level. KEM LVS, HKEM and to a lesser extent, the anato‐functional method perform well in maintaining the mean value of the PET‐unique regions, while reducing the white matter standard deviation (Fig. 12, top and middle rows). Figure 12 (bottom row) shows that the anato‐functional and MR‐guided methods lead to an increase in white matter mean value and decrease in gray matter mean values. This trend demonstrates that the anato‐functional and MR‐guided methods have lost their PVE reduction capabilities that were demonstrated in the high‐count real data studies (Fig. 10). Figure 13 shows the reconstructed images for each of the MR‐informed methods, with the parameters chosen to present an approximately fixed white matter standard deviation (as indicated by the arrowheads in Fig. 12).

## 4 Discussion

### 4.1 Simulation studies high count

The results from the high‐count simulation studies show that the anato‐functional method excels in concurrently improving the reconstruction of the whole brain and PET‐unique regions, when assessed via the majority of metrics presented in Figs. 3, 4, 5. The KEM LVS and HKEM also perform well in terms of the SSIM trade‐off (Fig. 3) between the PET‐unique and whole brain regions, and achieve a reduced white matter standard deviation for a fixed tumor mean value (Fig. 5). The Bowsher method achieves the lowest whole brain NRMSE (Fig. 3) and notably reduces PVEs, however it clearly suppresses the mean values of the PET‐unique regions (Fig. 5). The remaining MR‐informed methods are not the foremost method in respect to any of the presented metrics.

The superior performance of the anato‐functional method over HKEM can be attributed to the added flexibility provided by tuning the β regularization parameter. This is important due to the lack of PET structure present in the reconstructed PET image at low iterations, as such, the weighting factors (for the anato‐functional method) and basis functions (for HKEM) will be strongly influenced by the MR structure only, producing reconstructions similar to MR‐informed methods. Thus, a low level of regularization for the anato‐functional method is beneficial for high‐count datasets to prevent PET‐unique features being suppressed at low iterations. For HKEM, fewer voxels can be selected to contribute to the basis function (lower *k*NN) to prevent PET‐unique features being suppressed. As can be seen in Fig. 3 column 4, HKEM performs similarly to the anato‐functional method for *k*NN = 5, however for larger values of *k*NN the tumor regions are generally more biased. Therefore, the added flexibility of the regularised anato‐functional method improves its recovery of PET‐unique regions as well as the shared PET‐MR regions, in comparison with HKEM. Although for high regularization levels (high β), the anato‐functional methods will also suppress the PET‐unique regions.

Despite the beneficial attributes of the anato‐functional method over MR(only)‐informed methods, it should be noted that the inclusion of PET information for both HKEM and anato‐functional does degrade their noise reduction ability for regions with differing PET and MR structures as shown in the standard deviation images and bias vs standard deviation trade‐off plots for the PET‐unique regions (Fig. S2). In addition, the effective reduction in regularization that occurs for these PET‐MR‐informed methods at the PET‐unique regions may leave the corresponding reconstructed images more vulnerable to Gibbs like artefacts.^68^, ^69^ This is clearly a downside of the PET‐MR‐informed methods, however the increase in standard deviation (noise) is only observed in these particular regions (where the PET and MR structures differ) and at large β values. Both improvements in the noise reduction properties of PET‐MR‐informed methods, and more effective suppression of Gibbs like artefacts can be achieved through selecting a larger $\sigma_{\mathrm{PET}}$ parameter (as done for the low count simulations), resulting in broader PET similarity kernels. However, this will impair the discussed PET‐MR‐informed methods capability of reconstructing PET‐unique regions.

### 4.2 Low‐count simulation studies

The results from the low‐count simulation studies show that KEM LVS performs better than the other investigated methods in terms of the majority of metrics evaluated, such as NRMSE trade‐off, SSIM trade‐off (Fig. 6 columns 2 and 3) and tumor bias vs tumor standard deviation trade‐off (Fig. S3). The lack of dependence of KEM LVS on PET data helps to suppress the noise in the image, while the compactness of the basis functions enables improved recovery of the PET‐unique regions (relative to the other MR‐informed methods). HKEM also performs well when evaluated in terms of SSIM trade‐off, tumor bias vs whole brain standard deviation trade‐off (Fig. 6, columns 3 and 4), or tumor mean vs white matter standard deviation (Fig. 8). Both KEM and Bowsher methods lead to large reductions in whole brain NRMSE (Fig. 6, columns 2) and PVEs (Fig. 8, bottom row), however this is at the expense of suppression and possible deformation of the tumor regions (as shown in Fig. 7 and Fig. S3 bias‐std trade‐off and profiles). In contrast to the high‐count simulation studies, the anato‐functional method performs poorly in terms of the all the metrics presented (Fig. 6, 7, 8) for the low‐count simulation studies.

The noise present in the low‐count dataset clearly has a negative impact on the anato‐functional method, which fails to outperform the rival methods as it did for the high‐count dataset, (Figs. 6 vs 3). For the anato‐functional method, to preserve PET only edges requires the value of $\sigma_{\mathrm{PET}}$ to be relatively small. However, for noisy PET data, most of the PET voxel values will be quite different to the central voxel value, leading to most of the weighting factors going to zero. This limits the extent of regularization for the anato‐functional method resulting in noisy reconstructed images. This problem can be resolved by using a larger value of $\sigma_{\mathrm{PET}}$ (as chosen for the low count simulations), but this limits the influence of the PET update image, and hence the recovery of PET‐unique regions. By comparison, HKEM uses the *k*NN sparsification to select voxel to contribute to the basis function, based on their PET voxel value. The *k*NN sparsification allows PET features to be extracted, while allowing a large $\sigma_{\mathrm{PET}}$ value to be selected. Therefore, a fixed number of voxels are selected to contribute to each basis function, effectively fixing the level of regularization (irrespective of the noise present in the PET image). This allows HKEM to achieve an improved trade‐off in comparison to the anato‐functional method.

A further comparison of the high‐ and low‐count simulation studies (Figs. 4 vs 7) shows a major change in the MR‐guided (and anato‐functional) method's capability to delineate white and gray matter regions. This is due to the different $\sigma_{\mathrm{MR}}$ values selected (as shown in Table 1), for the different count levels. For the high counts dataset, a smaller value of $\sigma_{\mathrm{MR}}$ is selected (in comparison to the low count dataset value), emphasizing differences in MR voxel values, and thus enhancing edges in the reconstructed PET image. Whereas, for the low counts dataset, a larger value of $\sigma_{\mathrm{MR}}$ was selected, leading to similar weighting values for all MR voxels as differences in the MR voxels are effectively ignored, producing spatially smooth PET images. This trend is also observed for the high‐ and low‐count real data studies, where the same $\sigma_{\mathrm{MR}}$ parameters are used as in the corresponding simulation study.

### 4.3 Augmented FDG dataset

For the high‐count simulation and real data studies similar trends are observed. In both studies, the anato‐functional method followed by KEM LVS and HKEM perform better than the remaining MR‐informed methods when evaluated in terms of tumor mean vs white matter standard deviation trade‐off (Figs. 5 and 10). In particular, the anato‐functional method achieves the lowest white matter standard deviation for a fixed tumor mean for three out of the four tumor regions assessed (Figs. 5 and 10, top and middle row), while reducing PVEs with respect to postsmoothed MLEM (Figs. 5 and 10, bottom row). Therefore, the inclusion of PET information into the reconstruction process via the anato‐functional method (and HKEM) appears to be beneficial in the high‐count simulated and real patient datasets investigated. All MR‐informed methods investigated also demonstrate improved PVE correction for both simulation and real data studies, in comparison to postsmoothed MLEM. Visual support of the improved gray–white matter delineation is shown in Fig. 11.

For the low count simulation and real data studies, similar trends are also observed in terms of accurately reconstructing PET‐unique regions, while concurrently suppressing noise, with KEM LVS and to a lesser extent, HKEM performing better than the other methods investigated. In both simulated and real data studies, KEM LVS and HKEM achieve similar PVE reduction and noise suppression properties (Figs. 8 and 12, bottom row) to the Bowsher method, while reconstructing the PET‐unique regions with an increased mean value (Figs. 8 and 12, top and middle row) relative to Bowsher. The performance of the anato‐functional method, however, does differ slightly between the simulation and real data studies, with the anato‐functional method achieving an improved tumor mean vs white matter standard deviation trade‐off in the real data study (Figs. 8 vs 12). This change can be attributed to the $\sigma_{\mathrm{PET}}$ parameter, which was reselected for the real data studies. Excluding the anato‐functional method, a similar trend is observed between the simulated and real data studies for the remaining MR‐informed methods in terms of tumor mean vs white matter standard deviation (Figs. 8 and 12, top and middle row). The regional mean values for the MR‐informed methods also show similar results between the simulated and real data studies (Figs. 8 and 12, bottom row). In both cases, KEM provides the best reduction in PVEs (reducing the white matter mean and increasing the gray matter mean), whereas the anato‐functional and MR‐guided methods show the converse trend due to increased PVEs. The over‐smoothing of the MR‐guided and anato‐functional methods, in comparison with the other MR‐informed methods investigated (such as Bowsher) can be seen in Fig. 13.

## 5 Limitations

This study has used the image quality measures of NRMSE and SSIM to evaluate the proposed methods, which are useful for representing the performance of quantification tasks. However, the ability to interpret such error metrics as potential changes in patient diagnosis and management is limited. The ultimate evaluation of medical image quality is through task‐based observer studies, using either a human observer or a representative mathematical observer. Two popular means for undertaking such task‐based observer studies include receiver operating characteristic (ROC) curves and the use of the Hotelling observer.^70^, ^71^, ^72^, ^73^ For the presented comparison study, the use of human observers (radiologists in the case of PET‐MR images) would be very time‐consuming given the evaluation required of multiple patients and reconstruction methodologies. Ideally each scan would also be read by multiple radiologists to account for inter‐person variation in the reading of PET scans. In related work, a preliminary task‐based observer study has been undertaken, in which a reduced number of MR methods are evaluated for the diagnosis of Alzheimer's disease or temporal lobe epilepsy from reduced count PET images.^74^ Automating the task‐based observer process is still an area of active research within medical imaging, even for the relatively simple task of tumor detection.^75^, ^76^.

## 6 Conclusions

Three regularized and three reparameterized MR‐informed PET reconstruction methodologies have been compared. The capability of each method to reduce PVEs and the noise present in the reconstructed image without biasing the recovery of genuine high‐intensity PET‐MR mismatched regions was investigated.

For the high‐count dataset, the anato‐functional method provided the best SSIM and NRMSE whole brain to PET‐unique region trade‐off relative to the other methods investigated. The inclusion of the current PET image through the calculation of the weighting factors, as presented by the anato‐functional MAP method, enables PVE correction and noise suppression to be attained in regions of matching PET‐MR structure, while also reconstructing PET‐unique regions with a similar bias to unsmoothed MLEM.

For the low count simulated dataset, the use of spatially compact basis functions (KEM LVS) achieved the best SSIM and NRMSE trade‐off for the reconstruction of PET‐unique and whole brain regions, outperforming the other methods investigated. HKEM also performed well for the simulated and real datasets in terms of tumor mean vs white matter standard deviation, relative to the remaining MR‐informed methods. The presence of noise in the low count dataset (of similar intensity to the PET‐unique regions) leads to the inclusion of noise in the reconstructed PET image for the anato‐functional (and the HKEM to a lesser extent) method if certain parameters values were selected. Therefore, at low count levels the inclusion of PET information into the reconstruction process could be more beneficial if integrated in an alternative manner.

To conclude, for the reconstruction of noisy data, multiple MR‐informed methods produce favorable whole brain vs PET‐unique region trade‐off curves, very comfortably outperforming the whole brain denoising of postsmoothed MLEM, for a fixed PET‐unique region SSIM.

## Supporting information


**Appendix S1:** Appendix A: Comparison of KEM and KEM LVS basis functions.
Appendix B. Extended evaluation of simulated dataset.

**Fig. S1: **Basis functions derived using either the conventional kernel method (KEM) method or the KEM largest value sparsification (LVS) method. The impact of the different implementations on basis function shape is shown for a uniform (top row) and structured (bottom row) magnetic resonance (MR) region. Only the proposed KEM LVS can deliver compact basis functions in uniform MR regions and also structured basis functions in detail containing MR regions for the same fixed set of parameters.

**Fig. S2:** All the results shown are for the high counts (10^8^) simulated dataset, using multiple noise realizations. Bias Images: bias images for each of the positron emission tomography (PET)‐unique regions, shown for increasing level of β (MAP) or *k* (KEM) for each reconstruction method. Std Images: standard deviation images for each of the PET‐unique regions, shown for increasing level of β or *k* for each reconstruction method. Bias‐Std Trade‐off: Bias vs standard deviation plots for each of the PET‐unique regions, for increasing levels of β (MAP) or *k* (KEM) along each curve. Tumor Profiles: tumor profiles through the mean image (averaged across noise realizations) of each reconstruction method (approximately fixed normalized root mean square error), for each PET‐unique region.

**Fig. S3: **All the results shown are for the low counts (10^7^) simulated dataset, using multiple noise realizations. Bias Images: bias images for each of the positron emission tomography (PET)‐unique regions, shown for increasing level of β (MAP) or *k* (KEM) for each reconstruction method. Std Images: standard deviation images for each of the PET‐unique regions, shown for increasing level of β or *k* for each reconstruction method. Bias‐Std Trade‐off: bias vs standard deviation plots for each of the PET‐unique regions, for increasing levels of β (MAP) or *k* (KEM) along each curve. Tumor profiles: tumor profiles through the mean image of each reconstruction method (the selected parameters for which correspond to an approximately fixed whole brain normalized root mean square error, for each PET‐unique region. The maximum likelihood expectation maximization (MLEM) profile has been taken from the mean image of MLEM with post reconstruction smoothing applied (FWHM 3.5 mm).
