# Supplementary material for: Intercomparison of MR‐informed PET image reconstruction methods
Source: Med Phys. 2019 Oct 4;46(11):5055–74. doi: 10.1002/mp.13812 (PMC6899618; doi:10.1002/mp.13812)
Supplement: Supplementary file 1 — Appendix S1: Appendix A: Comparison of KEM and KEM LVS basis functions. Appendix B. Extended evaluation of simulated dataset. [file MP-46-5055-s001.pdf]

Appendix A: Comparison of KEM and KEM LVS Basis Functions

Figure A1 illustrates the differences between the KEM and KEM LVS basis functions when applied to a uniform MR region and a MR region with structure (detail). As shown in Fig. A1 only the KEM LVS basis function can achieve a compact basis function in the uniform region, whilst also fully extracting the MR structure in the detailed region, for the same fixed set of parameters.

Appendix B: Extended Evaluation of Simulated Dataset

For the high count (10^8) simulation studies, Fig. B1 shows for each tumour region: the bias images, standard deviation images, the bias vs standard deviation trade-off curves, and the tumour profiles through the mean image. For the tumour profiles, the selected parameters of each method correspond to a fixed whole brain NRMSE level shown in Fig.3 column 2. All error metrics in this figure are calculated from 10 noise realisations. In Fig. B1 (bias images and bias vs standard deviation plots), the MR(only)-informed methods (KEM, KEM LVS, Gaussian MR-Guided and Bowsher) can be seen to increasingly bias the PET-unique regions for increasing values of β or k, in comparison to un-smoothed MLEM. Through the incorporation of PET information into the weight calculation the PET-MR-informed methods, in particular the anato-functional method achieves a reduced bias for the more intense PET-unique regions B and D (Fig. B1, bias images), in comparison to the MR(only)-informed methods. For the bias standard deviation trade-off curves of the PET-unique regions B and D, the anato-functional method attains values similar to that of MLEM for the first five β values, the markers for which are therefore superimposed on top of one another. Consequently, the bias and standard deviation images of the anato-functional method have a very similar pattern to that of un-smoothed MLEM for the first five β values. These β values for the anato-functional method still lead to a reduction in the whole brain region NRMSE values, explaining the favourable NRMSE trade-off curves shown in Fig. 3 (columns 2 and 3). Considering the MR(only)-informed methods, the KEM LVS methods provides the best bias-standard deviation trade-off curves for three out of the four PET-unique regions, although the improvement is minimal and cannot be seen in the bias or standard deviation images.

For the low count (10^7) simulation studies, Fig. B2 shows for each tumour region: the bias images, standard deviation images, the bias vs standard deviation trade-off curves, and the tumour profiles through the mean image. For the tumour profiles, the selected parameters of each method correspond to a fixed whole brain NRMSE level shown in Fig.6 column 2. In this figure the KEM LVS method outperforms all other compared methods in terms

of the bias vs standard deviation plots for three out of the four PET unique regions (and provides the 2nd best bias vs standard deviation trade-off for the remaining PET unique region). The tumour profiles (bottom row) show the KEM LVS and HKEM method performing similarly to unsmoothed MLEM for the high intensity tumour regions B and D, whereas all MAP based methods result in a more supressed tumour profiles in comparison to MLEM.

Figure Captions:

Figure A1. Basis functions derived using either the conventional KEM method or the KEM LVS method. The impact of the different implementations on basis function shape is shown for a uniform (top row) and structured (bottom row) MR region. Only the proposed KEM LVS method can deliver compact basis functions in uniform MR regions and also structured basis functions in detail containing MR regions for the same fixed set of parameters.

Figure B1. All the results shown are for the high counts (10^8) simulated dataset, using multiple noise realisations. Bias Images: bias images for each of the PET-unique regions, shown for increasing level of β (MAP) or k (KEM) for each reconstruction method. Std Images: standard deviation images for each of the PET-unique regions, shown for increasing level of β or k for each reconstruction method. Bias-Std Trade-off: Bias vs standard deviation plots for each of the PET-unique regions, for increasing levels of β (MAP) or k (KEM) along each curve. Tumour Profiles: tumour profiles through the mean image (averaged across noise realisations) of each reconstruction method (approximately fixed NRMSE), for each PET-unique region.

Figure B2. All the results shown are for the low counts (10^7) simulated dataset, using multiple noise realisations. Bias Images: bias images for each of the PET-unique regions, shown for increasing level of β (MAP) or k (KEM) for each reconstruction method. Std Images: standard deviation images for each of the PET-unique regions, shown for increasing level of β or k for each reconstruction method. Bias-Std Trade-off: bias vs standard deviation plots for each of the PET-unique regions, for increasing levels of β (MAP) or k (KEM) along each curve. Tumour profiles: tumour profiles through the mean image of each reconstruction method (the selected parameters for which correspond to an approximately fixed whole brain NRMSE), for each PET-unique region. The MLEM

profile has been taken from the mean image of MLEM with post reconstruction smoothing applied (FWHM 3.5mm).
